# Extracorporeal membrane oxygenation for prevention of barotrauma in patients with respiratory failure: A scoping review

**DOI:** 10.1111/aor.14864

**Published:** 2024-09-21

**Authors:** Alessandro Belletti, Jacopo D’Andria Ursoleo, Enrica Piazza, Edoardo Mongardini, Gianluca Paternoster, Fabio Guarracino, Diego Palumbo, Giacomo Monti, Marilena Marmiere, Maria Grazia Calabrò, Giovanni Landoni, Alberto Zangrillo

**Affiliations:** ^1^ Department of Anesthesia and Intensive Care IRCCS San Raffaele Scientific Institute Milan Italy; ^2^ School of Medicine Vita‐Salute San Raffaele University Milan Italy; ^3^ Department of Health Science, Anesthesia and ICU School of Medicine, University of Basilicata San Carlo Hospital Potenza Italy; ^4^ Department of Cardiothoracic Anesthesia and ICU Azienda Ospedaliero‐Universitaria Pisana Pisa Italy; ^5^ Department of Radiology IRCCS San Raffaele Scientific Institute Milan Italy

**Keywords:** acute respiratory distress syndrome, extracorporeal membrane oxygenation, Macklin effect, mechanical ventilation, pneumomediastinum, pneumothorax

## Abstract

**Background:**

Barotrauma is a frequent complication in patients with severe respiratory failure and is associated with poor outcomes. Extracorporeal membrane oxygenation (ECMO) implantation allows to introduce lung‐protective ventilation strategies that limit barotrauma development or progression, but available data are scarce. We performed a scoping review to summarize current knowledge on this therapeutic approach.

**Methods:**

We systematically searched PubMed/MEDLINE, EMBASE, and the Cochrane Central Register of Controlled Trials for studies investigating ECMO as a strategy to prevent/limit barotrauma progression in patients with respiratory failure. Pediatric studies, studies on perioperative implantation of ECMO, and studies not reporting original data were excluded. The primary outcome was the rate of barotrauma development/progression.

**Results:**

We identified 21 manuscripts presenting data on a total of 45 ECMO patients. All patients underwent veno‐venous ECMO. Of these, 21 (46.7%) received ECMO before invasive mechanical ventilation. In most cases, ECMO implantation allowed to modify the respiratory support strategy (e.g., introduction of ultraprotective/low pressure ventilation in 12 patients, extubation while on ECMO in one case, and avoidance of invasive ventilation in 15 cases). Barotrauma development/progression occurred in <10% of patients. Overall mortality was 8/45 (17.8%).

**Conclusion:**

ECMO implantation to prevent barotrauma development/progression is a feasible strategy and may be a promising support option.

## Graphical abstract

Visual abstract presenting main article structure, objective, research methodology, and results.

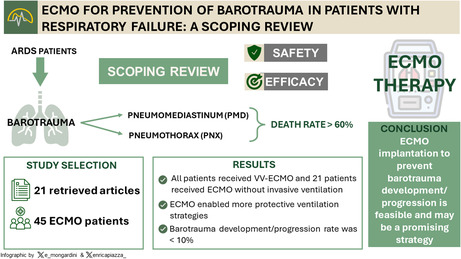

## 1 INTRODUCTION

Barotrauma is the term used to describe physical injury to body tissues produced by a pressure differential between a gas area inside the body or in touch with it and the fluid surrounding it.^1^ Pneumomediastinum (PMD) and pneumothorax (PNX) are typically interpreted as an indication of lung barotrauma.^2^

According to multiple studies, patients with acute respiratory distress syndrome (ARDS) often experience barotrauma—in the form of PMD and PNX—with a reported rate of occurrence ranging from 6% to 20%.^2^, ^3^ However, management of PNX/PMD in patients with respiratory failure is challenging and nonstandardized,^4^, ^5^, ^6^ and the death rate for ARDS patients who develop PNX/PMD may exceed 60%.^2^

Extracorporeal membrane oxygenation (ECMO) is a mechanical circulatory support device used to replace pulmonary gas exchange function or cardiac function in patients with severe respiratory or cardiovascular failure.^7^, ^8^, ^9^ In current practice, ECMO is generally regarded as a rescue device for the most severe cases who failed to respond to all other available support strategies.^7^, ^8^, ^10^, ^11^

Use of ECMO may be particularly attractive in patients with barotrauma, as the use of the membrane lung to ensure gas exchange could facilitate the institution of protective and ultraprotective ventilation,^12^ thus ultimately limiting pressures delivered to the airway/lung system. Of note, in some cases positive pressure invasive ventilation could potentially be avoided by the use of ECMO.^13^, ^14^ Accordingly, some authors hypothesized that limiting or avoiding at all positive pressure ventilation might either prevent the development of barotrauma or avoid its progression once barotrauma has occurred.^15^, ^16^ In a small case series of seven COVID‐19 patients with severe ARDS and at high risk for barotrauma, Paternoster et al. observed that early application of awake veno‐venous (V‐V)‐ECMO without invasive mechanical ventilation (IMV) resulted in low rates of intubation and death alongside no barotrauma occurrences.^17^ Nevertheless, paucity of data in the published literature on the use of ECMO to prevent or limit barotrauma exists.

Therefore, we performed a scoping review aiming to assess both the feasibility and efficacy of ECMO implantation in patients with or at risk for barotrauma to prevent its occurrence or further progression.

## 2 METHODS

Based on the guidelines from the Cochrane Collaboration and Centre for Reviews and Dissemination, we conducted a systematically structured scoping review in line with the Preferred Reporting Items for Systematic Reviews and Meta‐Analyses (PRISMA) checklist guideline and its extension for scoping reviews (PRISMA‐ScR).^18^ The PRISMA‐ScR checklist is included in the Supplementary Appendix (Supplementary Material).

The PICO (Patient/Population/Problem, Intervention, Comparison/Control, Outcome) approach was employed to formulate the review question: Among adult patients with or at risk for barotrauma (P), does the implantation of ECMO (I), compared to standard care (C), result in the prevention of barotrauma occurrence or in limiting its further progression (O)?

Our hypothesis was that ECMO implantation would allow to avoid invasive ventilation or maintain ultraprotective ventilation, which would in turn result in the prevention of barotrauma or the avoidance of its further progression.

### 2.1 Search strategy

Three experienced and independent investigators conducted a comprehensive, unbiased search on PubMed/MEDLINE, EMBASE, and the Cochrane Central Register of Controlled Trials databases from their inception to identify studies (up to May 10th, 2024, without inception limits) pertinent to the research question.

Details regarding the search strategy are made available in the Supplementary Appendix (Supplementary Material, Search Strategy).

Duplicate publications were removed using EndNote X9 (Clarivate Analytics), and the resulting citations were uploaded to Rayyan for screening.^19^

Notably, both backward and forward snowballing techniques were applied to scrutinize the references of selected articles, aiming to identify additional studies for potential inclusion in the systematic review.

No additional language restrictions were imposed.

### 2.2 Study selection

Following removal of duplicate records from multiple databases using Zotero duplicate identification and then manually checking deleted records, every reference identified through the database search and literature review underwent independent assessment by the three investigators, at both title and abstract levels. In cases where concerns or disagreements arose, full‐text articles were consulted, and any disagreements were resolved through discussion ultimately involving a third, senior investigator.

#### 2.2.1 Inclusion criteria

We used the following inclusion criteria: patients aged 18 years or older; with respiratory failure; with or at risk for barotrauma; undergoing ECMO implantation to prevent barotrauma occurrence or its further progression.

#### 2.2.2 Exclusion criteria

Studies concerning the pediatric population, studies on perioperative/periprocedural use of ECMO, publications not presenting original data (including narrative reviews, systematic reviews, meta‐analyses, commentaries, letters, and editorials), and works published in languages other than English for whose an English translation was not obtained were excluded from this review.

### 2.3 Data extraction and quality assessment

Two independent investigators conducted data extraction, aided by standardized forms for each of the included trials. All available data outlined in the research protocol, including study characteristics (such as first author, year of publication, and country), setting, sample size, details on ECMO support, and outcomes, were extracted.

#### 2.3.1 Risk of bias assessment

The risk of bias assessment was independently performed by two investigators with the Risk Of Bias In Non‐randomized Studies‐of Interventions (ROBINS‐I), as shown in the Supplementary Appendix (Supplementary Material, Table S2).^20^, ^21^ Disagreements were resolved during the review process by discussion with a third reviewer and by consensus. Based on this method, risk levels were classified as “high risk of bias,” “some concerns,” or “low risk of bias.” We considered an investigation as low risk of bias only if all domains were assessed as low risk of bias.

### 2.4 Primary outcome

The primary outcome of our study was the rate of barotrauma development or progression. Development of barotrauma was defined as development of PNX, PMD, or subcutaneous emphysema while on ECMO support. Progression of barotrauma was defined according to the authors of each individual study. If no definition was reported, barotrauma progression was defined as the need for additional therapeutic interventions to treat barotrauma (e.g. chest drain), or enlargement of original barotrauma (e.g. worsening PNX, development of bilateral PNX in a patient with unilateral PNX, development of PNX in addition to PMD, etc.).

Additional outcomes included all‐cause longest follow‐up mortality, successful weaning from ECMO/achievement of lung transplantation, and rate of intubation for patients receiving ECMO without invasive ventilation.

### 2.5 Statistical analysis

We presented the results from individual studies, typically encompassing predictive performance for predefined outcomes. Provided the heterogeneity in the literature and considering that most of the retrieved studies were case reports or case series with <5 patients, quantitative data synthesis or analysis were not performed.

## 3 RESULTS

**FIGURE 1:**
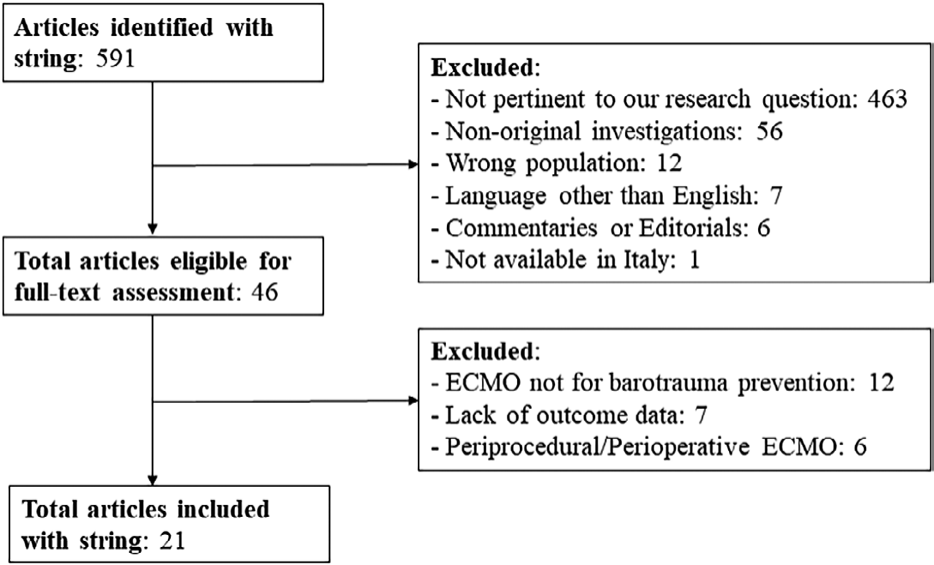
Flowchart of the studies selection and identification process.

Our search strategy identified 591 articles concerning the use of ECMO as a support strategy in patients with or at high risk for barotrauma. Of these, 544 studies were excluded after title and abstract assessment. One study was excluded because the full article was not available. Consequently, 46 studies were eligible for detailed assessment (Figure 1), of which 21 (enrolling a total of 45 ECMO patients) were subsequently selected for inclusion.^16^, ^17^, ^22^, ^23^, ^24^, ^25^, ^26^, ^27^, ^28^, ^29^, ^30^, ^31^, ^32^, ^33^, ^34^, ^35^, ^36^, ^37^, ^38^, ^39^, ^40^

The list of major exclusions with detailed reasons for exclusion is available in the Supplementary Appendix (Supplementary Material, Table S1).

### 3.1 Characteristics of the included studies

Details on study characteristics are presented in Table 1. All but three studies were published after 2017. The remaining three articles were published in 2009,^26^ 2015,^29^ and 2016,^36^ respectively. Four studies were performed in Japan,^23^, ^35^, ^38^, ^40^ three in the United States,^22^, ^30^, ^37^ two in China,^27^, ^39^ two in Germany^29^, ^34^ two in Italy,^17^, ^33^ and the others were published in Portugal,^32^ Saudi Arabia,^16^ Qatar,^36^ Ireland,^24^ Norway,^26^ UK,^28^ and Canada,^25^ respectively. Six were retrospective observational studies^17^, ^22^, ^25^, ^31^, ^32^, ^37^ and the remaining 15 were case reports. Only one study compared patients managed with an “ECMO‐first (invasive ventilation as rescue)” approach to patients managed with “invasive ventilation first (ECMO as rescue)” approach.^31^ Eleven studies investigated patients with Coronavirus Disease 2019 (COVID‐19) pneumonia/ARDS,^16^, ^17^, ^23^, ^25^, ^30^, ^31^, ^33^, ^34^, ^35^, ^38^, ^40^ three studies investigated patients with *P. jirovecii* pneumonia/ARDS,^32^, ^36^, ^39^ and two studies investigated patients with autoimmune‐related interstitial lung disease (i.e., dermatomyositis).^24^, ^27^ The remaining studies examined patients with mixed‐etiology ARDS,^37^ chest trauma‐related bronchopleural fistula,^22^ *Legionella pneumonia*/ARDS,^26^ Leptospirosis infection (with pulmonary hemorrhage),^28^ and non‐COVID‐19 pneumonia/ARDS.^29^

**TABLE 1:** Characteristics of included studies.

First author	Year	Country of origin	Study design	Setting	ECMO patients, no.	ECMO without invasive ventilation, no.	ECMO for barotrauma prevention or treatment
Ali HS^36^	2016	Qatar	Case Report	*P. jirovecii* Pneumonia/ARDS	1	0	Treatment in patients with established barotrauma
Alqatari S^24^	2018	Ireland	Case Report	Autoimmune‐related interstitial lung disease (dermatomyositis)	1	0	Treatment in patients with established barotrauma
Attou R^31^	2024	Belgium	Retrospective Observational/Cohort/Case series	COVID‐19 Pneumonia/ARDS	9 (plus 13 patients in the control group)	9	Treatment in patients with established barotrauma
Azzam MH^16^	2021	Saudi Arabia	Case Report	COVID‐19 Pneumonia/ARDS	1	1	Treatment in patients with established barotrauma
Barnacle J^28^	2020	UK	Case Report	Leptospirosis Infection (with pulmonary hemorrhage)	1	0	Treatment in patients with established barotrauma
El‐Battrawy I^29^	2015	Germany	Case Report	Non‐COVID‐19 Pneumonia/ARDS	1	0	Treatment in patients with established barotrauma
Golino G^33^	2024	Italy	Case Report	COVID‐19 Pneumonia/ARDS	1	0	Treatment in patients with established barotrauma
Grant A^22^	2020	USA	Retrospective Observational/Cohort/Case series	Chest trauma‐related (penetrating/blunt) bronchopleural fistula	3	0	Treatment in patients with established barotrauma
Gu Q^27^	2021	China	Case Report	Autoimmune‐related interstitial lung disease (dermatomyositis)	1	0	Treatment in patients with established barotrauma
Huang G^39^	2022	China	Case Report	*P. jirovecii* Pneumonia/ARDS	1	0	Treatment in patients with established barotrauma
Kishaba T^38^	2022	Japan	Case Report	COVID‐19 Pneumonia/ARDS	1	0	Treatment in patients with established barotrauma
Kohara J^35^	2022	Japan	Case Report	COVID‐19 Pneumonia/ARDS	1	0	Treatment in patients with established barotrauma
Nakatsutsumi K^40^	2020	Japan	Case Report	COVID‐19 Pneumonia/ARDS	1	0	Treatment in patients with established barotrauma
Odish MF^37^	2021	USA	Retrospective Observational/Cohort/Case series	ARDS, mixed etiology	4	0	Treatment in patients with established barotrauma
Paternoster G^17^	2022	Italy	Retrospective Observational/Cohort/Case series	COVID‐19 Pneumonia/ARDS	7	7	Prevention in high‐risk patients
Pereira SL^32^	2021	Portugal	Retrospective Observational/Cohort/Case series	*P. jirovecii* Pneumonia/ARDS	4	2	Prevention (3 patients) Treatment (1 patient)
Sekhon M^25^	2021	Canada	Retrospective Observational/Cohort/Case series	COVID‐19 Pneumonia/ARDS	3	0	Treatment in patients with established barotrauma
Takahashi S^23^	2023	Japan	Case Report	COVID‐19 Pneumonia/ARDS	1	0	Treatment in patients with established barotrauma
Thiara APS^26^	2009	Norway	Case Report	*Legionella* Pneumonia/ARDS	1	0	Treatment in patients with established barotrauma
Umlauf J^34^	2022	Germany	Case Report	COVID‐19 Pneumonia/ARDS	1	1	Treatment in patients with established barotrauma
Unold J^30^	2021	USA	Case Report	COVID‐19 Pneumonia/ARDS	1	1 (extubated while on ECMO)	Prevention in high‐risk patients

Abbreviations: ARDS, acute respiratory distress syndrome; COVID‐19, coronavirus disease 2019; ECMO, extracorporeal membrane oxygenation.

### 3.2 Extracorporeal membrane oxygenation and mechanical ventilation settings

Details on ECMO settings are presented in Table 2. All patients were treated with V‐V‐ECMO. Of these, 20 patients (44.4%) underwent ECMO implantation before receiving invasive ventilation. The most common cannulation configuration was femoro‐femoral, while heparin was the most commonly reported anticoagulant administered. Thirteen studies reported details on ventilation/respiratory support settings before and after ECMO implantation,^16^, ^23^, ^28^, ^29^, ^30^, ^31^, ^33^, ^35^, ^36^, ^37^, ^38^, ^39^, ^40^ and in all but one^31^ cases, ventilation settings were adjusted after ECMO implantation. In particular, patients were switched from conventional to ultraprotective ventilation in four studies (seven patients),^33^, ^35^, ^36^, ^37^ while lower peak inspiratory pressure (PIP) and positive end‐expiratory pressure (PEEP) were used in five studies (five patients).^23^, ^28^, ^38^, ^39^, ^40^ One patient was extubated while on ECMO.^30^ In one case,^29^ separate two‐lungs protective ventilation was used.

**TABLE 2:** Details on the different ECMO configurations, treatment duration, ventilator settings, and anticoagulation regimens.

First author	Year	ECMO configuration	Respiratory support before ECMO	Respiratory support during ECMO	Change in ventilation setting/respiratory support after ECMO initiation	Anticoagulation type	Initial cannulation configuration	ECMO duration (mean/median, days)
Ali HS^36^	2016	V‐V	IMV	IMV (ultraprotective)	Conventional to ultraprotective IMV	I.v. UFH	Fem‐fem	6
Alqatari S^24^	2018	N/A	IMV	IMV	N/A	N/A	N/A	N/A
Attou R^31^	2024	V‐V	HFNC	HFNC	No change in respiratory rate after ECMO implantation	I.v. UFH	Fem‐jug	N/A
Azzam MH^16^	2021	V‐V	HFNC	NIV + HFNC	Initiation of NIV on top of HFNC	I.v. UFH	Fem‐fem	18
Barnacle J^28^	2020	V‐V	IMV	IMV (ultraprotective)	Lower PIP and PEEP	I.v. UFH	Fem‐fem	8
El‐Battrawy I^29^	2015	V‐V	IMV	Separate two‐lungs protective ventilation	Switch to separate‐lungs ventilation	N/A	N/A	10
Golino G^33^	2024	V‐V	IMV	IMV (ultraprotective)	Conventional to ultraprotective IMV	I.v. UFH	Fem‐jug	12
Grant A^22^	2020	V‐V	IMV	IMV (ultraprotective EMPROVE protocol^41^, ^42^)	N/A	I.v. UFH	N/A	P1 = 24; P2 = 20; P3 = 16
Gu Q^27^	2021	V‐V	IMV	IMV	N/A	N/A	Fem‐jug	33
Huang G^39^	2022	V‐V	IMV	IMV	Lower PEEP	N/A	N/A	9
Kishaba T^38^	2022	V‐V	IMV	IMV	Lower PEEP and RR	S.c UFH	Fem‐fem	8
Kohara J^35^	2022	V‐V	IMV	IMV (ultraprotective)	Conventional to ultraprotective IMV	I.v. UFH	Fem‐fem	10
Nakatsutsumi K^40^	2020	V‐V	IMV	IMV (ultraprotective/very low pressures)	Very low PIP + ZEEP	I.v. UFH	Fem‐jug	10
Odish MF^37^	2021	V‐V	IMV	IMV (ultraprotective)	Conventional to ultraprotective IMV	N/A	Mixed	P1 = 25; P2 = 7; P3 = 12; P4 = 16
Paternoster G^17^	2022	V‐V	NIV + HFNC	NIV + HFNC	N/A	I.v. UFH	Mixed	15 (2–61)
Pereira SL^32^	2021	V‐V	IMV or COT	IMV or COT	N/A	N/A	N/A	P1 = 41; P2 = 12; P3 = 13; P4 = 26
Sekhon M^25^	2021	V‐V	IMV	IMV	N/A	N/A	N/A	N/A
Takahashi S^23^	2023	V‐V	IMV (high‐pressures)	IMV (protective ventilation pressures)	Lower PIP and PEEP	N/A	Fem‐jug	7
Thiara APS^26^	2009	V‐V	IMV (high‐pressures)	IMV	N/A	I.v. UFH	Fem‐jug	39
Umlauf J^34^	2022	V‐V	HFNC	HFNC	N/A	N/A	Fem‐jug	17
Unold J^30^	2021	V‐V	IMV	COT	Extubation while on ECMO	N/A	N/A	N/A

Abbreviations: COT, conventional oxygen therapy; ECMO, extracorporeal membrane oxygenation; HFNC, high‐flow nasal cannula; IMV, invasive mechanical ventilation; i.v., intravenous; N/A, not available; NIV, noninvasive ventilation; PEEP, positive end‐expiratory pressure; PIP, peak inspiratory pressure; s.c., subcutaneous; UFH, unfractionated heparin; V‐V, veno‐venous; ZEEP, zero end‐expiratory pressure.

### 3.3 Primary and secondary outcomes

#### 3.3.1 Barotrauma development in high‐risk patients

Three studies (11 patients, 31.4%) reported “prophylactic” use of ECMO in patients at risk for barotrauma,^17^, ^30^, ^32^ while in all other cases ECMO was implanted after barotrauma development.

Criteria to define high‐risk of barotrauma were: (i) presence of Macklin‐like radiological sign^15^, ^43^, ^44^ on baseline chest computed tomography;^17^ (ii) presence of large emphysematous bullae;^30^ and (iii) *P. jirovecii* pneumonia.^32^

Overall, one patient (1/11; 9.1%) among those undergoing “prophylactic” ECMO developed barotrauma (asymptomatic pneumothorax),^30^ while two patients died (2/11; 18.2%).^17^

#### 3.3.2 Barotrauma progression in patients with barotrauma at the time of extracorporeal membrane oxygenation implantation

A total of 34 patients (75.6%) presented barotrauma at the time of ECMO implantation. In only two cases^24^, ^27^ (2/24, 8.3%), there was a worsening of the initial barotrauma following support with ECMO, while six patients died (17.6%).^24^, ^31^ Of note, one of these patients was among those exhibiting barotrauma progression,^24^ while for the others no data on barotrauma progression was available.

#### 3.3.3 Extracorporeal membrane oxygenation versus invasive ventilation

Only one study reported data comparing an “ECMO‐first” versus an “invasive ventilation first” approach for patients with COVID‐19 ARDS and pneumomediastinum.^31^ The authors did not report data on barotrauma progression but reported lower mortality rates in patients receiving an “ECMO‐first” approach (55% versus 92%). Of note, 55% of the “ECMO‐first” patients ultimately required invasive ventilation, while 61% of the “invasive ventilation first” patients required ECMO support. All of the patients requiring escalation of support died.

#### 3.3.4 Secondary outcomes

Overall, 36 patients (80%) were weaned off ECMO or underwent lung transplantation. A total of eight (17.8%) patients died,^17^, ^24^, ^31^ while the remaining patient was still receiving ECMO support when the original study was published.^25^

Among patients undergoing ECMO without invasive ventilation, need for intubation occurred in six patients (6/21, 28.6%).

Further details on outcomes are presented in Table 3.

**TABLE 3:** Primary and secondary outcomes.

First author	Year	Barotrauma development (for patients receiving ECMO for prevention), no.	Barotrauma progression (for patients receiving ECMO for treatment), no.	ECMO weaning/LTx achieved, no.	Need for intubation for patients on ECMO w/o IMV, no.	Longest follow‐up mortality, no.
Ali HS^36^	2016	N/A	0	1	N/A	0
Alqatari S^24^	2018	N/A	1	0	N/A	1
Attou R^31^	2024	N/A	N/A	4	5	5
Azzam MH^16^	2021	N/A	0	1	0	0
Barnacle J^28^	2020	N/A	0	1	N/A	0
El‐Battrawy I^29^	2015	N/A	0	1	N/A	0
Golino G^33^	2024	N/A	0	1	N/A	0
Grant A^22^	2020	N/A	0	3	N/A	0
Gu Q^27^	2021	N/A	0	1	N/A	0
Huang G^39^	2022	N/A	0	1	N/A	0
Kishaba T^38^	2022	N/A	0	1	N/A	0
Kohara J^35^	2022	N/A	0	1	N/A	0
Nakatsutsumi K^40^	2020	N/A	0	1	N/A	0
Odish MF^37^	2021	N/A	0	4	N/A	0
Paternoster G^17^	2022	0	N/A	5	1	2
Pereira SL^32^	2021	0	0	4	0	0
Sekhon M^25^	2021	N/A	0	2	N/A	0
Takahashi S^23^	2023	N/A	0	1	N/A	0
Thiara APS^26^	2009	N/A	1	1	N/A	0
Umlauf J^34^	2022	N/A	0	1	0	0
Unold J^30^	2021	1	N/A	1	0	0

Abbreviations: ECMO, extracorporeal membrane oxygenation; IMV, invasive mechanical ventilation; LTx, lung transplantation; N/A, not available.

## 4 DISCUSSION

### 4.1 Key findings

In this scoping review, we found that ECMO implantation with the goal of limiting barotrauma progression is feasible and is also generally associated with good outcomes, although data remains scarce and generally limited to individual case reports. We also found that colleagues generally consider ECMO implementation to allow ultraprotective and/or very low‐pressure ventilation, while in almost half of the reported cases an “ECMO without invasive ventilation” approach was selected. Our data mirror our original hypothesis.

### 4.2 Relationship to previous studies

Our scoping review aimed to systematically assess the current practice on ECMO use for preventing barotrauma occurrence or limiting its progression. Previous reviews either investigated the effect of ECMO on survival, the feasibility and safety of ECMO without invasive ventilation, or the feasibility and safety of physiotherapy on ECMO.^9^, ^13^, ^45^, ^46^ Compared with these reviews, our study focused on a very specific patient population. We found a greater rate of successful weaning from ECMO and survival than the one reported for the general ARDS population on ECMO,^9^ as well as for ARDS patients with barotrauma.^2^ However, this is likely explained by the fact that studies included in our review present data of a highly selected population treated in experienced centers. Furthermore, studies reporting unsuccessful outcomes are less likely to be published. Nevertheless, we cannot exclude that the low mortality rate observed in our study may at least in part be related to the efficacy of the investigated strategy. Notably, our rate of awake ECMO failure is in line with what has already been reported in the published literature for patients with ARDS.^13^

Previous randomized controlled trials comparing ultraprotective ventilation with standard protective ventilation strategies in patients with extracorporeal support did not report data on barotrauma,^47^, ^48^ or found no difference in its occurrence rate between ultraprotective and standard protective ventilation.^49^ Compared with these studies, our review focused on patients with or at high risk for barotrauma, therefore focusing on a highly selected population representing 5 to 15% of patients generally enrolled in ARDS trials.^2^, ^3^ Furthermore, a relevant proportion of our patients were COVID‐19 patients, who are considered to be at higher risk for barotrauma as compared with non‐COVID‐19 ARDS patients.^2^, ^50^, ^51^

Previous systematic reviews on the management of air leaks during mechanical ventilation confirmed that the general approach of critical care clinicians includes ventilation strategies aimed at reducing airway pressures, a finding also confirmed by our study.^4^, ^5^ Compared with these studies, which only briefly mentioned the possibility of using ECMO, we specifically focused on the possibility of ECMO implantation to facilitate either ultraprotective invasive ventilation with very low airway pressure or avoidance of positive pressure ventilation at all.

### 4.3 Implication of study findings

Our study provides baseline data on the current practice and patient outcomes on use of ECMO to prevent barotrauma development and progression in patients with respiratory failure. Our data suggest that ECMO implantation in this setting is feasible and potentially associated with good outcomes. Our data showed that the general approach of clinicians is to implant ECMO in order to allow for ultraprotective and/or low‐pressure ventilation. Both results are in line with our original hypothesis. Notably, in about half of the reported cases, clinicians chose to avoid invasive ventilation at all while on ECMO, suggesting that some colleagues begin to consider this as a viable alternative approach to ultraprotective ventilation. In one additional case report, the patient was extubated while on ECMO.^30^ These strategies were generally associated with either avoidance of barotrauma progression or development in retrieved studies. Only one before/after retrospective study compared an “ECMO‐first” to an “invasive ventilation first” approach for COVID‐19 ARDS patients with barotrauma and found that the “ECMO first” approach might be associated with improved survival.^31^ Notably, in this study, all patients requiring escalation of support died, confirming the high mortality associated with barotrauma development and/or failure of awake ECMO in ARDS patients.^2^, ^13^

One additional study used a well‐known radiological sign (the Macklin‐like radiological sign or Macklin effect) to identify patients with severe COVID‐19 ARDS at high‐risk for barotrauma and candidate these patients to the “ECMO first” approach while avoiding invasive ventilation.^17^ The Macklin effect has been associated with a very high risk of the development of barotrauma in COVID‐19 ARDS patients,^52^, ^53^, ^54^, ^55^, ^56^ and some authors suggested applying ECMO without invasive ventilation to prevent barotrauma in these high‐risk patients,^15^, ^17^ either using an “ECMO first” approach or extubating patients while on ECMO.^57^

The use of ECMO without invasive ventilation is a well‐established practice in patients awaiting lung transplantation,^13^, ^58^ and became increasingly popular also for adult and pediatric patients with COVID‐19.^13^, ^59^, ^60^ The principal advantages of awake ECMO include prevention of issues associated with sedation and immobilization, improved communication with relatives and staff, and avoidance of complications related to invasive ventilation such as ventilator‐associated pneumonia.^14^, ^45^, ^46^, ^61^ The present study offers preliminary evidence to support the hypothesis that awake ECMO may also be effective in the treatment or prevention of barotrauma, supporting the hypotheses of some authors.^15^, ^17^, ^62^

It is noteworthy that some authors have also reported the complete avoidance of ventilation while on ECMO to prevent ventilation‐associated lung injury in patients with such severely depressed lung compliance that even ultraprotective ventilation becomes unfeasible.^63^ This approach may prove an interesting alternative for the management of such extreme conditions.

Of note, most of the studies included in our review focused on COVID‐19 patients. The pathophysiology of COVID‐19 ARDS is different from non‐COVID‐19 ARDS,^64^, ^65^, ^66^, ^67^ and therefore our results may not apply to non‐COVID‐19 patients.

Collectively, our data suggested that the use of ECMO to prevent or limit barotrauma progression may indeed warrant further investigations, and we provide some baseline data to plan future studies. In particular, our study highlighted that “awake” ECMO without invasive ventilation is a relatively common approach in this setting, the other being ECMO alongside ultraprotective ventilation. Future studies should compare these strategies with current standard care to assess feasibility, safety, and efficacy on a wider scale of each approach and investigate different populations.

### 4.4 Study limitations

Our study has some limitations. The limited number of patients enrolled contributes to the heterogeneity of the findings; hence, our investigation needs to be considered hypothesis‐generating only. However, this remains the largest review on the topic available to date.

The fact that the majority of included investigations are in the form of case reports underscores that, at present, the use of ECMO for barotrauma prevention remains anecdotal. However, management of an air leak in the context of severe respiratory failure is challenging, and very few data are available to guide its therapeutic management.

Most studies investigated patients with COVID‐19 ARDS; therefore, our findings may not be generalized to reflect other populations of critically ill patients.

Only one study included a control group undergoing invasive ventilation without ECMO; therefore, there is very limited data on direct comparison with other approaches.

## 5 CONCLUSIONS

In this scoping review, we found that ECMO implantation to prevent or limit barotrauma progression in patients with respiratory failure is feasible and may be associated with good patient outcomes. However, available data remain sparse and mostly limited to individual case reports and COVID‐19 ARDS patients. The most commonly used approaches are ECMO without invasive ventilation or ECMO with ultraprotective invasive ventilation.

## AUTHOR CONTRIBUTIONS

**Alessandro Belletti**: Conceptualization, Methodology, Investigation, Data curation, Formal analysis, and Writing—Original Draft. **Jacopo D'Andria Ursoleo**: Conceptualization, Methodology, Investigation, Data curation, Formal analysis, and Writing—Original Draft. **Enrica Piazza**: Resources, Methodology, Data curation, and Writing—Review and Editing. **Edoardo Mongardini**: Resources, Methodology, Data curation, and Writing—Review and Editing. **Gianluca Paternoster:** Investigation, Data curation, and Writing—Review and Editing. **Fabio Guarracino**: Investigation, Data curation and Writing—Review and Editing. **Diego Palumbo:** Investigation, Data curation, and Writing —Review and Editing. **Giacomo Monti:** Conceptualization, Methodology, Resources, and Writing—Review and Editing. **Marilena Marmiere:** Resources, Methodology, Data curation, and Writing—Review and Editing. **Maria Grazia Calabrò:** Validation, Supervision, Methodology, and Writing—Review and Editing. **Giovanni Landoni**: Validation, Supervision, Methodology, and Writing—Original Draft. **Alberto Zangrillo**: Validation, Supervision, Methodology, and Writing—Review and Editing.

## FUNDING INFORMATION

Funded by the European Union ‐ Next Generation EU ‐ NRRP M6C2 ‐ Investment 2.1 Enhancement and strengthening of biomedical research in the NHS (Project code: PNRR‐MAD‐2022‐12376796; CUP code: C43C22001330007).

## CONFLICT OF INTEREST STATEMENT

None.

## ETHICS APPROVAL AND CONSENT TO PARTICIPATE

Not applicable.

## Supporting information


Appendix S1.

## Data Availability

Further information is available from the corresponding author upon reasonable request.
