# Supplementary material for: Extracorporeal membrane oxygenation for prevention of barotrauma in patients with respiratory failure: A scoping review
Source: Artif Organs. 2024 Sep 21;49(2):183–95. doi: 10.1111/aor.14864 (PMC11752986; doi:10.1111/aor.14864)
Supplement: Supplementary file 1 — Appendix S1. [file AOR-49-183-s001.docx]

**Supplementary Material**

***Extracorporeal Membrane Oxygenation for Prevention of Barotrauma in Patients with Respiratory Failure: A Scoping Review***

*A. Belletti, J. D’Andria Ursoleo, E. Piazza, E. Mongardini, D. Palumbo, G. Paternoster,*

*F. Guarracino, G. Monti, M. Marmiere, M.G. Calabrò, G. Landoni, A. Zangrillo*

**Contents**

PubMed Search Strategy p. 2

Supplementary Table S1 p. 3

Supplementary Table S2 p. 5

**PubMed Search strategy**

("Extracorporeal Membrane Oxygenation"[MeSH Terms] OR "ecmo"[Text Word] OR "ecmo"[Title/Abstract] OR "vaecmo"[Text Word] OR "vaecmo"[Title/Abstract] OR "ecls"[Text Word] OR "ecls"[Title/Abstract] OR "va ecmo"[Text Word] OR "va ecmo"[Title/Abstract] OR "extracorporeal*"[Text Word] OR "extracorporeal*"[Title/Abstract] OR "extra corporeal*"[Text Word] OR "extra corporeal*"[Title/Abstract] OR (("extracorporeal*"[Text Word] OR "extracorporeal*"[Title/Abstract] OR "extra corporeal*"[Text Word] OR "extra corporeal*"[Title/Abstract]) AND ("membrane*"[Text Word] OR "membrane*"[Title/Abstract]) AND ("oxygenat*"[Text Word] OR "oxygenat*"[Title/Abstract])) OR (("extracorporeal*"[Text Word] OR "extracorporeal*"[Title/Abstract]) AND ("life"[Text Word] OR "life"[Title/Abstract]) AND ("support"[Text Word] OR "support"[Title/Abstract] OR "supports"[Text Word] OR "supports"[Title/Abstract]))) AND ("emphysema"[Title/Abstract] OR "emphysema"[Text Word] OR "barotrauma"[Title/Abstract] OR "barotrauma"[Text Word] OR "pneumothorax"[Text Word] OR "pneumothorax"[Title/Abstract] OR "pneumomediastinum"[Title/Abstract] OR "pneumomedistinum"[Text Word])

**Supplementary Table S1.** List of the major exclusions with reasons for exclusion.

| **First Author** | **Year** | **Journal** | **PubMed ID** | **Exclusion criteria** |
| --- | --- | --- | --- | --- |
| A. W. Martinelli | 2020 | *European Respiratory Journal* | 32907891 | Lack of outcome data |
| A. Shah | 2023 | *Perfusion Journal* | 36877783 | Lack of outcome data |
| K. Miwa | 2022 | *Yonago Acta Medica* | 36474899 | Periprocedural/Perioperative ECMO |
| K. Momii | 2021 | *Medicine Journal* | 33663129 | ECMO not for barotrauma prevention |
| S. Besa | 2021 | *Respiratory Medicine Case Reports* | 34341715 | ECMO not for barotrauma prevention |
| H. He | 2017 | *Critical Care Medicine* | 28728546 | Lack of outcome data |
| B. E. Garfield | 2023 | *Critical Care Medicine* | 37276353 | Lack of outcome data |
| B. Ficial | 2023 | *Journal of Clinical Medicine* | 36835885 | Lack of outcome data |
| Y. Toyoda | 2021 | *Journal Trauma Acute Care Surgery* | 34238854 | Lack of outcome data |
| M. Yanada | 2008 | *Interactive Cardiovascular and thoracic surgery* | 18757451 | Periprocedural/Perioperative ECMO |
| N. Tsurumachi | 2023 | *Minerva Anestesiologica* | 37432315 | Periprocedural/Perioperative ECMO |
| J. Rubin | 2022 | *Chest Imaging and Pathology for Clinicians* | 35131063 | ECMO not for barotrauma prevention |
| C. Yu | 2022 | *Medicine Journal* | 35089240 | ECMO not for barotrauma prevention |
| J. Huang | 2019 | *Medicine Journal* | 31096495 | ECMO not for barotrauma prevention |
| T. Horii | 2020 | *Radiology Case Report* | 32989407 | ECMO not for barotrauma prevention |
| M. Koeppen | 2017 | *Case Reports in Anesthesiology* | 28523194 | Lack of outcome data |
| T. Yamada | 2014 | *Acute Medicine & Surgery* | 29930831 | Periprocedural/Perioperative ECMO |
| N. Madershahian | 2007 | *Journal of Cardiac Surgery* | 17488410 | ECMO not for barotrauma prevention |
| Y. S. Wu | 2012 | *Transplant Infectious Disease* | 23279741 | ECMO not for barotrauma prevention |
| Y.T. Lin | 2009 | *Journal of Chinese Medical Association* | 19762318 | Periprocedural/Perioperative ECMO |
| P. Y. Brichon | 2012 | *Interactive Cardiovascular and thoracic surgery* | 22761125 | ECMO not for barotrauma prevention |
| N. Shaikh | 2021 | *Qatar Medical Journal* | 34722222 | ECMO not for barotrauma prevention |
| Y. Ishikawa | 2020 | *General Thoracic and Cardiovascular Surgery* | 32845450 | Periprocedural/Perioperative ECMO |
| Y. Kondo | 2014 | *Critical Care* | 25042680 | ECMO not for barotrauma prevention |
| E. Bassi | 2011 | *Revista Brasilera de Terapia Intensiva* | 23949411 | ECMO not for barotrauma prevention |

**Supplementary Table S2.** Risk of bias assessment in case series/observational studies.

| **First Author** | **Bias due to confounding** | **Bias in selection of participants into the study** | **Bias in classification of interventions** | **Bias due to departures from intended exposures** | **Bias due to missing data** | **Bias in measurement of outcomes** | **Bias in selection of the reported result** | **Overall bias** |
| --- | --- | --- | --- | --- | --- | --- | --- | --- |
| Attou R | Low | Low | Low | Low | Low | Low | Low | Some concerns |
| Grant A | Some concerns | Low | Low | Low | Some concerns | Low | Some concerns | Some concerns |
| Odish MF | Some concerns | Low | Low | Low | Some concerns | Low | Low | Some concerns |
| Paternoster G | Some concerns | Low | Low | Low | Low | Some concerns | Low | Some concerns |
| Pereira SL | Some concerns | Low | Low | Low | Some concerns | Low | Some concerns | Some concerns |
| Sekhon M | No information | No information | No information | No information | No information | No information | No information | Unclear |
